# A Case of Lethal Abdominal Compartment Syndrome due to Rapidly Expanding Ovarian Small Cell Carcinoma Pulmonary Type

**DOI:** 10.7759/cureus.9879

**Published:** 2020-08-19

**Authors:** Eric M Sieloff, Michael Baumgartner, Mark Schauer, Brandy Shattuck

**Affiliations:** 1 Internal Medicine, Western Michigan University Homer Stryker M.D. School of Medicine, Kalamazoo, USA; 2 Internal Medicine , Western Michigan University Homer Stryker M.D. School of Medicine, Kalamazoo, USA; 3 Pathology, Western Michigan University Homer Stryker M.D. School of Medicine, Kalamazoo, USA

**Keywords:** abdominal compartment syndrome, ovarian cancer, cardiac arrest

## Abstract

A 53-year-old woman presented with a rapidly growing pelvic mass suspected to be endometrial cancer due to endometrial biopsy showing grade 1 endometrioid adenocarcinoma. Due to severe aortic valve stenosis, she underwent a transcatheter aortic valve replacement (TAVR) for surgical optimization for a planned total abdominal hysterectomy, bilateral salpingo-oophorectomy, and tumor debulking. She was discharged on dual antiplatelet therapy with plans for future surgery, but was readmitted with abdominal distension, constipation, and urinary retention. The pelvic mass seen on prior imaging studies had increased in size. Unanticipated asystole cardiac arrest occurred two days after readmission, which on autopsy was found to be secondary to abdominal compartment syndrome from a rapidly enlarging ovarian small cell carcinoma pulmonary type.

## Introduction

Compartment syndrome occurs in a confined space, when increased pressure leads to organ dysfunction as the result of ischemia. Many surgeons are familiar with compartment syndrome in the extremities following trauma, but it can also occur in the abdomen [1]. The World Society on Abdominal Compartment Syndrome defines intra-abdominal hypertension (IAH) as an intra-abdominal pressure (IAP) greater than 12 mmHg, and abdominal compartment syndrome (ACS) as a sustained IAP greater than 20 mmHg that is associated with new organ dysfunction/failure [2].

While most cases of primary IAH result from peritonitis, pancreatitis, and inflammatory or oncologic diseases, other causes include aortic aneurysm, abdominal and retroperitoneal tumors, mechanical bowel obstruction, abdominal trauma, and ascites [3]. Secondary causes include obesity, large volume fluid resuscitation, and sepsis [4]. ACS has many systemic effects, including decreased renal and hepatic portal blood flow, resulting in decreased cardiac output and decreased lung and chest wall compliance [2].

In this case report, we discuss the unusual presentation of ACS secondary to an extremely rare gynecological malignancy, which led to our patient’s eventual demise. Early recognition and management can be crucial to reducing morbidity and mortality in cases of ACS.

## Case presentation

A 53-year-old woman presented to the emergency department with a 12-month history of menometrorrhagia and a one-week history of worsening nausea and vomiting. Her past medical history included a moderate developmental delay, severe valvular aortic stenosis, insulin-dependent diabetes mellitus, hypertension, morbid obesity, and hypothyroidism. She had no smoking history. On physical examination, the patient was 216 lbs with a BMI of 51.9 lbs/in^2^ and a blood pressure at 162/72 mmHg while tachycardic at 102 beats per minute. A 4/6 aortic systolic murmur was auscultated, and her abdomen was distended with mild tenderness to palpation, particularly in the lower quadrants. She was found to have a stage 3 acute kidney injury with a creatinine of 5.7 mg/dL (baseline 1.4 mg/dL), calcium of 9.3 mg/dL, lactic acid of 1.2 mmol/L, hemoglobin of 7.8 g/dL, and leukocytosis with a white blood cell count of 65,000/mm^3^. A CT scan of her abdomen and pelvis showed a 15.5-cm pelvic mass with areas of high attenuation noted within and extending into the adjacent ascites. A review of previous imaging (CT urogram from nine months prior) showed no previous evidence of these findings. A CT of her chest was unremarkable. 

Her aortic valve area was measured to be 0.49 cm^2^ six months prior on an echocardiogram and precluded her from receiving a dilation and curettage for her menometrorrhagia. Cardiac intervention with a transcatheter aortic valve replacement (TAVR) was planned but had not yet been accomplished.

The patient was admitted, and a paracentesis removing 3 L was performed. The serum ascites albumin gradient was <1.1 g/dL with a protein level of 3.5 g/dL and an albumin of 1.90 g/dL, and ascites cytology showed no malignant cells. A CT scan of her abdomen and pelvis and a transvaginal ultrasound showed a heterogeneous mass in the pelvis, superior and to the right of the uterus measuring 17.3 x 15.9 x 11.3 cm; these radiological findings were thought to be “likely consistent with a large focus of hemorrhage/hematoma” or an undetermined malignancy. The uterus had a 10-mm endometrial stripe, the right ovary measured 5.0 x 6.8 x 3.4 cm and the left ovary measured 3.3 x 1.8 x 2.4 cm.

The patient was cognitively impaired due to developmental delay that prevented her from being able to make informed medical decisions. Due to a complicated social situation, it took nearly a week to establish her sister as her legal designated power of attorney (DPOA) that unfortunately delayed her treatment. A gynecology oncology consultant recommended an endometrial biopsy and resection of her pelvic mass by a total abdominal hysterectomy, bilateral salpingo-oophorectomy, and tumor debulking. Anesthesiology and cardiology consultants recommended cardiac optimization for this surgery, which included a TAVR procedure.

The CA-125 (cancer-antigen 125) was found to be significantly elevated at 1,357 U/mL. An endometrial tissue biopsy was performed for which a pathology review determined it to be an endometrioid adenocarcinoma, Federation of Gynecology and Obstetrics (FIGO) grade 1. A bilateral uterine artery embolization was performed to mitigate her menometrorrhagia, during which a consultant interventional radiologist noted her uterus to be hypervascular with equal blood supply from bilateral uterine arteries along with “an enlarged right ovarian artery arising from the right uterine artery supplying the enhancing portions of the previously seen large pelvic mass.” This enlarged ovarian artery was also embolized. This was the first radiographic evidence that her pelvic mass may have been of ovarian origin.

Cardiology and cardiothoracic surgery performed a TAVR after a delay of several days due to scheduling issues. The patient was initiated on dual antiplatelet therapy with aspirin and clopidogrel with plans for one month of uninterrupted therapy, after which her surgery would be performed. A multidisciplinary meeting was arranged with consultants from radiology, gynecology oncology, medical oncology, and pathology for future treatment planning. It was concluded that (1) her pelvic mass most closely resembled a bulky uterine or ovarian malignancy with possible mesenteric implants, and no further biopsies were recommended due to the patient’s need for continual dual antiplatelet therapy; and (2) recommended preoperative treatment was megestrol acetate, as grade 1 endometrial adenocarcinoma is typically a hormonally responsive tumor, and the patient and her sister had chosen not to pursue any neoadjuvant chemotherapy. Recommendations for adjuvant chemotherapy or additional treatment would be based on final surgical pathology.

A leukocytosis is persistent throughout her admission, which was suspected to be secondary to tumor production of granulocyte colony stimulating factor (G-CSF); this has been documented in the medical literature to be associated with ovarian cancers. Her acute kidney injury resolved and her creatinine decreased to 1.18 mg/dL. Two paracenteses performed during her admission removed a total of 4 L. After a three-week hospital admission, she was discharged to a skilled nursing facility on her previous diuretic therapy, aspirin and clopidogrel for her aortic valve replacement, and megestrol acetate for her endometrial cancer with a close gynecology oncology follow-up for surgical planning.

**Figure 1:**
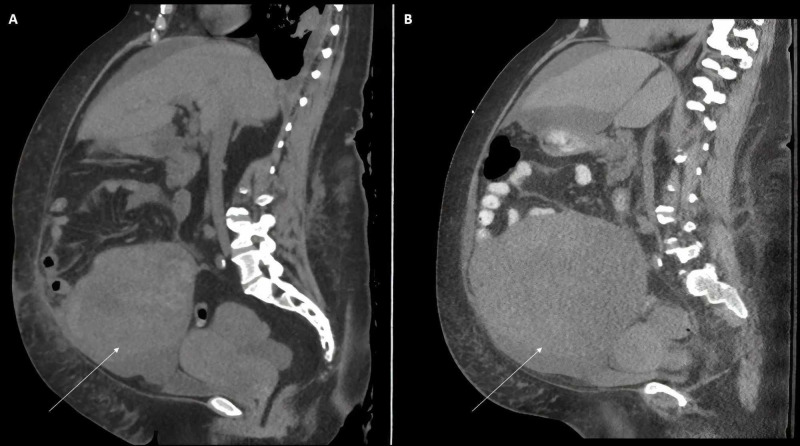
A rapidly growing pelvic mass, initially (A) and at readmission three weeks later (B). Arrow: pelvic mass

Two days after her discharge, the patient returned to the emergency department with dyspnea, lower extremity pitting edema, worsened abdominal distension, significant constipation, and newly reported urinary retention. Her weight had increased to 240 lbs, a 24-lb increase since at her previous admission. She was afebrile with a blood pressure of 103/68 mmHg, yet tachycardic to 112 beats per minute and tachypneic to 24 respirations per minute. She appeared comfortable and was oxygenating appropriately on ambient air. Her abdomen appeared slightly more distended without any tenderness or peritoneal signs, and she denied any significant abdominal pain. She continued to have a leukocytosis of 44,000/mm^3 ^and her creatinine was 1.26 mg/dL with a hemoglobin of 8.6 g/dL; there were no other significant lab findings. There was no evidence of any active infection and her lactic acid was 1.9 mmol/L. A foley catheter was placed, and 150 mL of urine was removed. An abdominal ultrasound showed only minimal abdominal ascites (an insufficient amount for a paracentesis) with an inaccessible ascites pocket in the pelvis along with a pelvic mass that appeared larger than during previous admission, confirmed by a CT scan (Figure 1).

Cardiology was consulted along with gynecology oncology to coordinate her surgery as soon as possible, as her new symptoms were suspected to be secondary to mass effect from her pelvic tumor.

On the second hospital day, the patient was evaluated in the morning and reported no new symptoms other than difficulty sleeping. Her vital signs had stabilized overnight, and she reported no significant abdominal pain, though she continued to be oliguric despite a urinary catheter placement. Two hours later, she suffered an asystole cardiac arrest and expired despite attempts at cardiopulmonary resuscitation.

**Figure 2:**
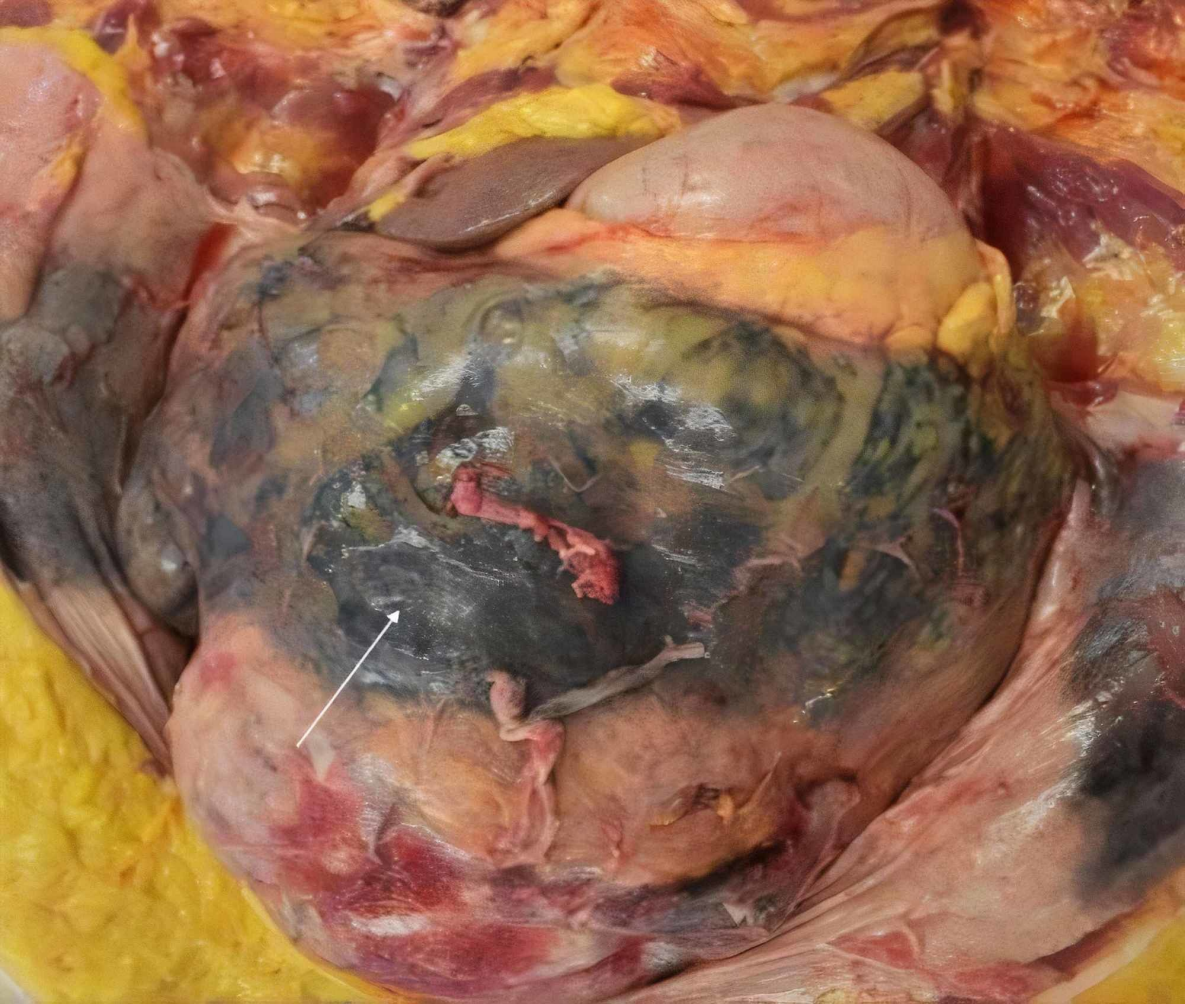
Ischemia of the abdominal mesentery. Arrow: ischemic mesentery.

The patient’s sister requested an autopsy to further investigate for a cause of death and the primary medical team suspected a pulmonary embolism occurred due to her sudden demise, despite having received adequate subcutaneous heparin deep vein thrombosis (DVT) prophylaxis. During the autopsy, when incising the abdomen, approximately 2 L of blood-tinged fluid was ejected under high pressure. The patient’s diaphragm was found to be displaced upwards by abdominal contents and a large pelvic mass, around which there was a focal discoloration of the bowel and mesentery suggestive of ischemia (Figure 2).

**Figure 3:**
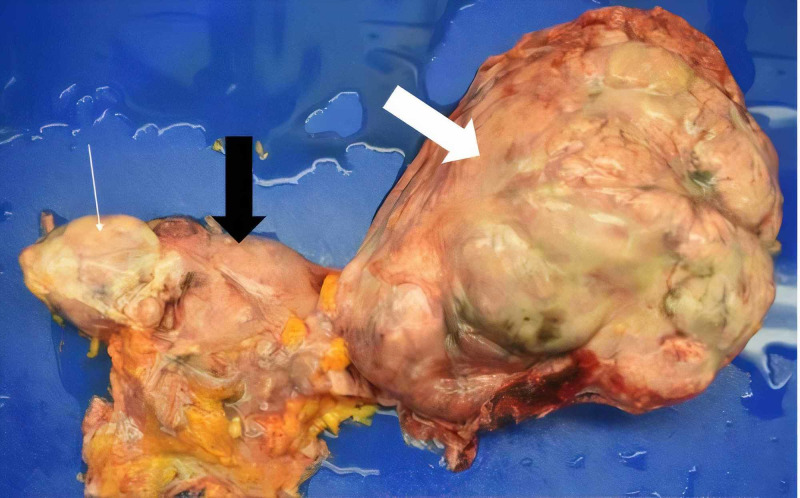
Left ovary, uterus, and large right ovary. Thin white arrow: left ovary. Thick black arrow: uterus. Thick white arrow: right ovary.

No thromboembolisms were found. The necrotic endometrium was found to be 0.1-0.5 cm in thickness and the right ovary measured 19 x 14.2 x 12 cm with a 10-cm central cavity containing 500 mL of bloody necrotic fluid; both uterine arteries were embolized in addition to an enlarged ovarian artery branching off the right uterine artery, which had resulted in this central necrosis. The left ovary was 6 x 8 x 4.2 cm. The massive right ovary was somewhat unexpected as on previous imaging it could not be clearly distinguished due to pelvic organs being so adherent and surrounded by abdominal ascites (Figure 3).

**Figure 4:**
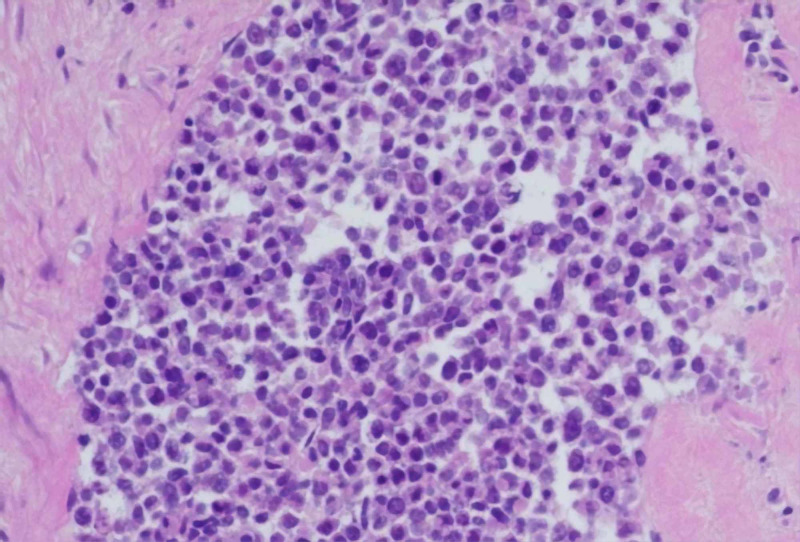
Hematoxylin and eosin (H&E) staining at ×200 showing round blue cells with hyperchromatic nuclear chromatin and mitotic figures arranged in a sheet.

On microscopic examination, the endometrium was found to be diffusely necrotic secondary to artery embolization and tissue from the right ovary appeared as small round blue cells with hyperchromatic chromatin on microscopy, most closely resembling ovarian small cell carcinoma of the pulmonary type (OSCCPT) (Figure 4).

With OSCCPT being a rare malignancy, at the time of this report there is no established immunohistochemical profile and the diagnosis of OSCCPT was made based on the combination of histological pattern, clinical presentation, and supportive immunohistochemical staining. It was determined that a rapidly growing OSCCPT tumor and its associated ascites had resulted in ACS causing this patient’s untimely demise.

## Discussion

We herein present a case of lethal ACS secondary to OSCCPT, a gynecological malignancy of which only a few cases have been reported in the literature [5]. Throughout this patient’s hospital course, it was difficult to assess and interpret this patient’s abdominal exam given her obese body habitus. We did not suspect ACS as she continued to deny abdominal pain and bloating, despite the rapidly enlarging pelvic mass. Conflicting interpretations of imaging studies added to the confusion. We suspect that with a large mass in her pelvis and elevated IAPs, a positional change may have led to the complete collapse of her inferior vena cava resulting in her asystole cardiac arrest.

IAH and ACS are related entities with the former being a sustained IAP > 12 mmHg, and the latter being a sustained IAP > 20 mmHg with new organ dysfunction/failure [2]. The abdominal pressures are determined by measuring intravesical pressures using a specialized bladder catheter while a patient is laying in a horizontal position, a procedure performed only in the ICU at our institution when our patient did not warrant an ICU admission. ACS can be caused by numerous factors, most commonly abdominal surgery, major trauma, hemoperitoneum, acute pancreatitis, and massive fluid resuscitation [6]. The patient tends to be critically ill, but if alert often conveys feelings of abdominal pain and bloating. A physical exam often reveals a distended abdomen and oliguria/anuria can be present in addition to pulmonary decompensation. Emergent surgical decompression of the abdomen is the treatment of choice to relieve the increased IAP [7].

Rapidly expanding intra-abdominal malignancies are an uncommon cause of ACS, and there are only a few cases of such in medical literature. In children, these malignancies have included neuroblastomas, nephroblastomas, and immature teratomas [8,9]. In adults, these primary tumors are often gynecological in nature, primarily benign ovarian cystadenomas with additional case reports of an ovarian granulosa cell tumor and a Burkitt lymphoma of the small intestine [10-15].

Ovarian malignancies can have several unique characteristics as were demonstrated in this case: they can be associated with significant leukocytosis and can produce massive amounts of ascites. A review by Viau and colleagues suggests that, though uncommon, the presence of a gynecologic malignancy with a leukocytosis in the absence of infection should prompt suspicion for a phenomenon known as paraneoplastic leukemoid reaction, which is suspected to be secondary to autonomous secretion of G-CSF hematopoietic growth factor and IL-6 [16]. Evidence by Thacker and colleagues and Shameem and colleagues demonstrates that the presence of G-CSF can stimulate growth in bladder cancer cells and osteosarcoma tumors, and thus autonomous factor production in gynecological malignancies could explain such rapid expansion and leukocytosis [17,18]. Malignant ascites can be seen with ovarian cancers, suspected to be the result of tumor cells producing excessive amounts of ascites while obstructing the lymphatic drainage system. A diagnostic paracentesis can be performed in an attempt to identify malignant cells from the ascitic fluid, in addition to tissue biopsy [19]. A single center study by Krugmann and colleagues reviewed 191 cases of ovarian cancer that revealed high-grade serous papillary ovarian cancers often had malignant cells present in the ascitic fluid, while the opposite was seen with neuroendocrine tumors including small cell carcinoma of the ovary, hypercalcemic type [20].

## Conclusions

We present a case of a patient with endometrial adenocarcinoma and a rapidly growing OSCCPT with malignant ascites and a paraneoplastic leukemoid reaction whose surgery was delayed in order to establish a DPOA and complete post-TAVR dual antiplatelet therapy. She was readmitted to the hospital at which time there was no clinical suspicion for ACS in the absence of abdominal pain, lactic acidosis, or any significant organ dysfunction. It was only when her post-mortem autopsy was performed that the cause of her death was established to be ACS causing her cardiac arrest to the dismay of her medical team. The patient’s outcome was affected by unfortunate delays in therapy due to the delay from her TAVR procedure being performed, the need to establish a legal DPOA, the uncertainty of diagnosis of her pelvic mass on imaging studies, and the need for 30 days of uninterrupted dual antiplatelet therapy following her TAVR before performing surgery. A high clinical index of suspicion is necessary to diagnose ACS, and this should be considered in a distended abdomen with a rapidly expanding intra-abdominal tumor.
